# Risk factors for poor treatment outcomes among opioid-dependent clients taking methadone in Mombasa, Kenya

**DOI:** 10.3389/adar.2024.11791

**Published:** 2024-06-07

**Authors:** Nassoro Mwanyalu, Maria Nunga, Raphael Mwanyamawi, Saade Abdallah, Maurice Owiny

**Affiliations:** ^1^ Department of Health Service, Mombasa County, Mombasa, Kenya; ^2^ Field Epidemiology and Laboratory Training Program, Ministry of Health, Nairobi, Kenya; ^3^ Consultant, Mombasa, Kenya

**Keywords:** methadone, HIV infections, treatment outcome, opioids, Hepatitis C, viral load, lost-to-follow-up

## Abstract

**Background:** The Methadone Maintenance Treatment (MMT) program has been proven to be beneficial in reducing illicit opioid use, increasing access to and retention of HIV treatment and other therapies, and reducing HIV transmission, and other drug-related morbidities and mortalities. However, determinants of treatment retention and outcomes for opioid-dependent persons accessing MMT in Kenya are limited. We sought to identify factors contributing to poor treatment outcomes among opioid-dependent persons enrolled in the Mombasa MMT program, between 2017 and 2019.

**Method:** We conducted a retrospective records review for opioid-dependent persons receiving Methadone treatment in the Kisauni MAT clinic enrolled during 2017–2019. We defined poor clinical or health-related treatment outcome as any client Lost-To-Follow-Up (LTFU), turned HIV or Viral hepatitis positive, and/or missed two or more antiretroviral therapy (ART) appointments intake during MMT. Variables abstracted from clinical and pharmacological MMT service delivery tools included socio-demographic characteristics, clinical history, risk factors, and MMT outcomes. Data were analyzed using *Epi Info7*. We calculated Prevalence Odds Ratios (POR) and 95% Confidence Intervals (CI) to identify factors associated with adverse health outcomes.

**Results:** Of the total 443 eligible records, the mean age was 37 years (SD ± 7.2) and males comprised 90.7%. The majority of females clients, 79.1% (34/43), were aged ≤35 years, 7.0% (3/43) had no education, 32.6% (14/43) were employed, 39.5% (17/43) were HIV positive and 18.6% (8/43) were HCV-positive. Overall, adverse treatment outcomes were at 27.5% (122/443), namely: LTFU at 22.8% (101/443), new HIV cases at 1.0% (4/391), HCV at 1.2% (5/405), and Hepatitis B Virus (HBV) at 1.2% (5/411), and 1.1% (5/443) died. Of HIV-infected clients linked to Comprehensive Care Clinic (CCC), 3.6% (2/56) defaulted from ART, and 25% (2/8) had detectable Viral Load of those retested. Lack of formal education (POR: 2.7, 95% CI: 1.3–5.7), unemployment (POR: 2.4, 95% CI: 1.4–4.0), and being a Non-Injector (POR: 1.7, 95% CI: 1.0–2.9) were negatively associated with treatment retention.

**Conclusion:** Females were younger, and more educated with higher HIV and HCV prevalence. Being a Non-injector, unemployment, and lack of formal education may increase the likelihood of poor treatment outcomes among MMT clients. Closer monitoring of MMT clients with these characteristics is recommended with the integration of CCC into MMT services.

## Introduction

Illicit substance use disorder is a global problem with over 15 million opioid-dependent people, and more than 70% of drug deaths are associated with opioid use [1]. Other adverse consequences of opioid use include a higher risk of HIV and viral hepatitis, drug overdose, and criminality [2, 3]. The World Health Organization recommends Methadone Maintenance Treatment (MMT) also called Medically Assisted Therapy (MAT) program, the use of an approved drug (such as Methadone, Buprenorphine, and Naltrexone) in combination with counselling and behavioural therapies, for the treatment of Opioid Use Disorder (OUD) [4, 5]. Documented evidence shows MMT to be beneficial in reducing illicit opioid use, increasing access to and retention of HIV treatment and other therapies, reducing HIV transmission rates, and other drug-related morbidity and mortality issues [4, 6–8].

Kenya has an estimated 0.16%–1.3% prevalence of opioid use disorder among people aged 15–64 years [1]. To forestall the spread of HIV and related co-morbidities among opioid-dependent persons, Kenya’s Ministry of Health (MOH), through the National AIDS and STI Control Program (NASCOP), embraced the MAT program in 2014. The medication of choice was Methadone [9, 10]. With half of the estimated people who inject drugs (PWID) dwelling in the Kenyan coastal counties, Mombasa and Kilifi counties were prioritized for MMT initiation in 2015 [10–12]. While various studies conducted in Kenya on people with opioid use disorder mostly focused on the period prior to MMT enrolment [13–15], a few studies have enquired about its effect on polydrug use, other related risky behaviour, productivity, household income food insecurity, and the economic cost of methadone [12, 16–18].

Mombasa County is among the six coastal counties located along drug trafficking routes in the southeastern part of the former Coast Province of Kenya bordering the Indian Ocean. The first MMT clinic in Mombasa, Coast General Teaching and Referral Hospital (CGTRH), was closed in 2016 within a year of its operation due to a doctor’s strike. All the CGTRH-enrolled MMT clients were subsequently transferred to a newly established Kisauni MAT clinic to continue their treatment. Its closer proximity to drug consumption spaces increased the demand among target clients, thereby exceeding the set MMT target. Despite evidence of continued drug overdoses, increased HIV and Hepatitis C Virus (HCV) infections, and frequent attrition among MMT clients [19, 20], the Kisauni MAT clinic had maintained a high number of MMT clients as of May 2019. Therefore, the study aimed to assess treatment outcomes within 2 years and identify factors associated with Lost-To-Follow-Up (LTFU) among MMT clients in Mombasa, Kenya.

## Materials and methods

### Design and setting

**FIGURE 1:**
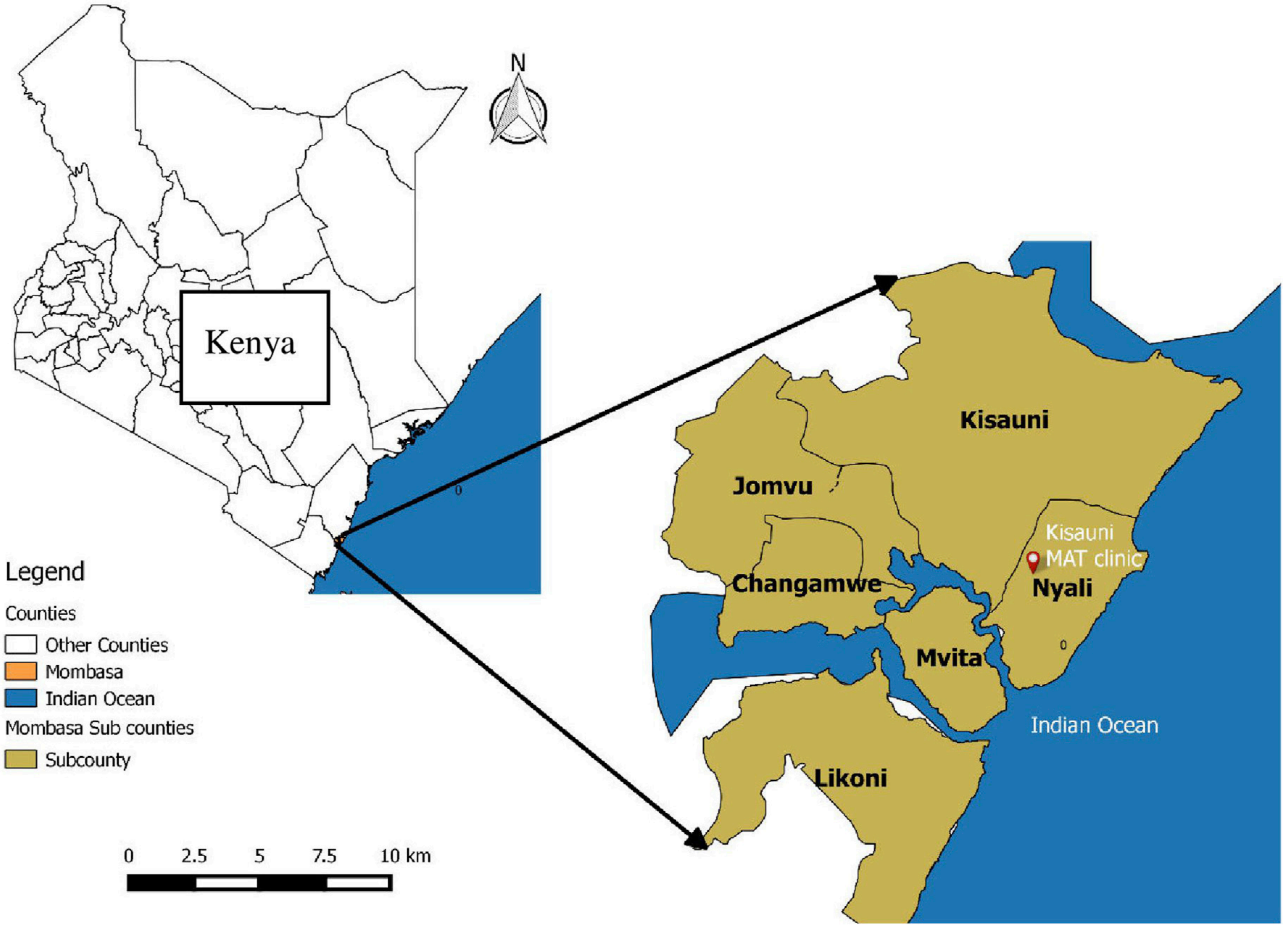
Map of Kenya showing location of Kisauni MAT clinic in Mombasa County, Kenya.

Mombasa County, located along the Kenyan coastal line, is the country’s tourism centre, with 68% of wage employment arising from tourism activities hosts the Kisauni MAT clinic (Figure 1). The Kisauni MAT clinic is an outpatient public health facility offering a package of MMT services for opioid-dependent patients in the county in collaboration with two Civil Society Organizations, the Muslim Education and Welfare Association (MEWA), and Reach Out Centre Trust (RCT).

The CSOs identify potential MMT clients from their drop-in centre (DIC) who undergo pre-screening for drug use and infectious disease before being linked to the program. Clients found to be eligible start methadone treatment after providing consenting to MMT regulations, which include supervised daily methadone dose intake, regular attendance of psychosocial support sessions, and periodic urine drug screening. The Methadone Maintenance Treatment (MMT) program at the clinic has four stages that ensure comfort and safety: 1) **Induction Stage:** low methadone dose initiation with gradual dose optimization; 2) **Stabilization Stage:** Which lasts 4–6 weeks to achieve therapeutic effects through dose adjustments; 3) **Maintenance Stage:** Retains clients on a steady methadone dose with regular clinical and psychological reviews; and **Cessation Stage:** Recommended after 12–24 months of MMT and undertaken only upon client’s request. It involves gradual methadone tapering and often requires family support. The CSOs also trace clients who drop out of MMT and provide psychosocial support during their re-induction [9, 21].

### Study design

We conducted a cross-sectional study at the Kisauni MMT clinic in Mombasa, Kenya for opioid-dependent persons enrolled in 2017–2019. Retrospective records review was performed through abstraction of clinical, laboratory and psychosocial data. Clients enrolled during 2017–2019 were eligible while those who transferred into or out of the clinic or had been in MMT for less than 3-month were excluded. The study was carried out in October and December 2019.

### Data collection tools and variables

We abstracted data from individual patient files, MAT Client Cards, paper-based and MS Excel MAT enrolment sheets, laboratory, pharmacy, and clinical registers including antiretroviral therapy (ART) registers. During data collection, we first extracted data from existing MAT MS Excel Sheets and verified them against data captured in MAT Client Cards and individual patient files. We later reviewed and checked the laboratory, ART, and pharmacy registers.

A data abstraction tool was used to capture variables during the records review. The key variables of interest extracted were categorized as Socio-demographic characteristics: (Age, sex, occupation, marital status), Drug Use History: (modes of drug use, routes of drug administration at induction, duration of opioid use before MAT), Clinical History: [HIV status, Hepatitis B Virus (HBV)], HCV and Treatment outcomes: MMT Outcomes [Retention, Weaned off, Defaulter, Lost to follow up (LTFU), Died] HIV Treatment (Viral Load, adherence). The national MAT protocol defines an MMT Defaulter as any client missing at least five consecutive daily methadone doses while LTFU was any MMT client who had missed 30 or more consecutive daily methadone doses. The national ART treatment protocol recommends 30-day prescriptions ART monthly pickup, whether obtained at the facility or offsite. We defined poor clinical and health-related treatment outcomes as any client who was an LTFU or acquired HIV, HBV or HCV while on MMT or missed ≥2 monthly ART pickups and or client who died while on MMT, regardless of the cause of death.

### Data analysis

Following data cleaning and verification, descriptive and analytic data analysis were conducted. Frequencies, proportions and ratios were computed for categorical data and mean, and median were generated for continuous variables. To determine HIV, HBV or HCV acquisition, we divided the number of new cases identified during MMT by the number of susceptible clients retested.

We used bivariate analysis with prevalence odds ratios (POR) as the measures of association to identify factors associated with LTFU. Statistically significance of the association was determined using the Chi-squared and Fisher exact test. We considered, factors whose POR were at ≥95% confidence level, to be associated with LTFU.

### Ethical considerations

Ethical approval was not required for the study involving humans in accordance with the local legislation and institutional requirements. Written informed consent to participate in this study was not required from the participants or the participants' legal guardians/next of kin in accordance with the national legislation and the institutional requirements. Permission to access MMT records was obtained from the office of the Sub-County Medical Officer of Health, and the Kisauni MAT facility-in-charge For data collection, only information that was relevant to the objectives of the study was extracted. To assure confidentiality, we anonymized client information by generating unique IDs.

## Results

### Social-demographic characteristics

Of the 457 records reviewed, 443 met the inclusion criteria. The majority, (90.3%, 400/443), were male clients, with females comprising only 9.7% (43/443) of the eligible clients. The overall mean age was 37 years SD (±7.2), 44.7% (198/443) of all clients were between 36 and 45 years and females aged ≤35 years were 79.1% (34/43). Slightly less than a third of the clients (30.7%, 136/443), were married. Interestingly, the distribution of marital status was fairly consistent across genders, with 30.1% of males and 34.9% of females reporting being married. On education level, a significant majority (76.1%, 337/443), had formal education; 27.9% (11/43) of females versus 15% (61/400) had post post-primary level of education. Men accounted for 45.3% (181/400) of those in employment, while women made up 32.6% (14/43) (Table 1).

**TABLE 1:** Baseline characteristics of enrolled clients for methadone maintenance treatment clients, at Kisauni MAT clinic.

Variable	Female (*n* = 43) count (%)	Male (*n* = 400) count (%)	Total (*N* = 443) count (%)
Age group			
≤25	11 (25.58)	24 (6.00)	35 (7.90)
26–35	23 (53.49)	133 (33.25)	156 (35.21)
36–45	8 (18.60)	190 (47.50)	198 (44.70)
46–55	1 (02.33)	53 (13.25)	54 (12.19)
Education			
None	3 (6.98)	30 (7.50)	33 (7.45)
Primary	28 (65.12)	309 (77.25)	337 (76.07)
Secondary	11 (25.58)	57 (14.25)	68 (15.35)
Post Secondary	1 (2.33)	4 (1.00)	5 (1.13)
Marital status			
Single	18 (41.86)	144 (36.00)	162 (36.57)
Married	15 (34.88)	121 (30.25)	136 (30.70)
Separated	6 (13.95)	36 (9.00)	42 (9.48)
Divorced	1 (2.33)	30 (7.50)	31 (7.00)
Widowed		5 (1.25)	5 (1.13)
Unknown	3 (6.98)	64 (16.00)	67 (15.12)
Employment Status			
Employed	14 (32.56)	181 (45.25)	195 (44.02)
Unemployed	18 (41.86)	129 (32.25)	147 (33.18)
Unknown	11 (25.58)	90 (22.50)	101 (22.80)

### Drug use and clinical history

Of the clients enrolled on the MMT program, at baseline, 98.1% (434/443) were mixing heroin and other drugs and 28.4% (126/443) were categorized as injecting drug users (IDUs) while the rest were non-injectors. The median duration of opioid use was 6 years (IQR 5–12) and 23.1% (102/443) had used heroin for less than 5 years prior to MMT enrollment. Concurrent usage of methadone and other substances during MMT was at 15.8% (70/443), with those taking cannabis contributing the most at 65.6% (48/70) (Table 2).

**TABLE 2:** Drug use and clinical history of enrolled clients at Kisauni MAT clients, 2017–2019 (*N* = 443).

Variable	Female (*n* = 43) Count (%)	Male (*n* = 400) Count (%)	Total (*N* = 443) Count (%)
Period of drug use before MMT			
≤5	16 (37.21)	100 (25.00)	116 (26.19)
6–15	24 (55.81)	254 (63.50)	278 (62.75
≥16	3 (06.98)	46 (11.50)	49 (11.06)
Mode of opioid use at enrolment			
Injectors	17 (39.53)	109 (27.25)	126 (28.44)
Non-injectors	26 (60.47)	291 (72.75)	316 (71.56)
Illicit drug use during MMT			
No	37 (86.05)	336 (84.00)	373 (84.20)
Yes	6 (13.95)	64 (16.00)	70 (15.80%)
Type of illicit drug used during MMT (*n* = 70)			
Bhang	4 (09.30)	44 (11.00)	48 (10.84)
Benzodiazepines	4 (09.30)	25 (06.25)	29 (06.55)
Tricyclic antidepressant	4 (09.30)	14 (03.50)	18 (04.06)
Heroin		7 (01.75)	7 (01.58)
Methamphetamine		6 (01.50)	6 (01.35)
Phencyclidine (PCP)		3 (0.15)	3 (00.68)
HIV status at Baseline			
Negative	26 (60.47)	365 (91.25)	391 (88.26)
Positive	17 (39.53)	35 (8.75)	52 (11.74)
Hepatitis B Virus at baseline			
Negative	38 (88.37)	373 (93.25)	411 (92.78)
Positive	5 (11.63)	27 (06.75)	32 (07.22)
Hepatitis C Virus at baseline			
Negative	35 (81.40)	367 (91.75)	402 (90.74)
Unknown		2 (00.50)	2 (00.45)
Positive	8 (18.60)	3 1 (07.75)	38 (8.81)

Overall HIV prevalence was 13.1% (58/443), compared to HBV prevalence 7.2% (32/443), and HCV 8.8% (39/443). Females were disproportionately vulnerable to all the diseases with 39.53% (17/43) HIV prevalence versus 8.75% for males, HBV and HCV prevalence of 11.63% (5/43), and 18.6% (8/43), respectively (Table 2).

All HIV-infected clients (58/58) were enrolled in a Comprehensive Care Clinic (CCC) across the county with only 13.8% (8/58) accessing ART at the Kisauni clinic. Of the eight clients taking their ART at the clinic, 75% (6/8) had an Undetected Viral Load.

### Treatment outcomes

During the study period, 75% (333/443) of MMT clients were Active, 22.8% (101/443) were Lost to Follow Up, 1.1% (5/443) had died during MMT and only 1.0% (4/443) of all clients had successfully weaned off. A higher proportion of female MMT clients (32.5%, 14/43) were LTFU.

One in four or 27.5% (122/443) of all enrolled MMT clients experienced poor clinical and health-related treatment outcomes while on MMT. These include 1.0% (4/391) who acquired HIV infection, 1.2% (5/411) who became HBV positive, and 1.2% (5/405) HCV positive. Other MMT negative outcomes were antiretroviral therapy interruptions at 3.6% (2/56), LTFU to MMT at 22.8% (101/443) and those who died at 1.1% (5/443) (Table 3).

**TABLE 3:** Reported poor treatment outcomes among enrolled MMT clients in Kisauni MAT clinic (*n* = 122).

Variable	Male frequency (%)	Female frequency (%)	Cumulative frequency (%)
Lost-to-Follow-up	87 (71.31)	14 (11.48)	101 (82.79)
HIV Positive during MAT	4 (3.28)		4 (3.28)
HBV Positive during MAT	4 (3.28)	1 (0.82)	5 (4.27)
HCV Positive during MAT	5 (4.27)		5 (4.27)
Died during MAT	5 (4.27)		5 (4.10)
Defaulter to ART during MAT	1 (0.82)	1 (0.82)	2 (1.71)

### Factors associated with lost to follow up (LTFU)

Computation of prevalence Odds ratios revealed a statistically significant association of the various factors with being Lost to Follow Up, namely,: Lack of formal education (POR 2.7, CI: 1.3–5.7), unemployment (POR: 2.4, 95% CI: 1.4–4.0), and being a Non-Injector (POR: 1.7, 95% CI: 1.02–2.9). The period of drug use before joining MMT, polysubstance use during MMT, type of illicit drug used while taking methadone, and living with HIV throughout MMT were all assessed but found to be non-significant (Table 4).

**TABLE 4:** Factors associated with poor treatment outcomes among opioid-dependent clients enrolled for methadone maintenance treatment (MMT), Kisauni MAT clinic, 2017–2019.

Variable	LTFU	Retention to MMT	POR (CI)	*p*-value
Gender				
Female	14	29	1.7 (0.9, 3.3)	0.18
Male	87	313		
Age at enrolment in years				
≤25	9	26	1.2 (0.54, 2.6)	0.40
≥25	92	316		
Education status				
Not educated	14	19	2.7 (1.3, 5.7)	0.005
Educated	87	323		
Employment Status				
Unemployed	56	91	2.4 (1.4, 4.0)	0.003
Employed	40	155		
Mode of drug use at enrolment				
Non-Injector	80	237	1.7 (1.0, 2.9)	0.03
Injector	21	105		
Illicit drug use during MMT				
Yes	14	56	0.8 (0.4, 1.5)	0.3
No	87	286		
Years of Opioid Use before joining MMT				
>5	68	259	0.7 (0.4, 1.1)	0.6
≤5	33	63		
Tricyclic antidepressant				
Positive	4	14	0.9 (0.25, 2.6)	0.9
Negative	87	280		
Bhang				
Positive	7	41	0.5 (0.2, 1.2)	0.5
Negative	84	253		
Benzodiazepines				
Positive	5	24	0.7 (0.2, 1.7)	0.4
Negative	86	270		
Heroin				
Positive	2	5	1.3 (0.2, 6.7)	0.8
Negative	89	289		
Methamphetamine				
Positive	1	6	0.5 (0.02, 3.7)	0.6
Negative	90	289		
HIV infection				
Positive	12	44	0.9 (0.5, 1.8)	0.47
Negative	89	298		

## Discussion

Our analysis of the MMT data revealed that females were younger, more educated, and had higher HIV, HBV, and HCV prevalence compared to males. We also found a higher MMT retention rate and undetected viral loads among the MMT clients living with HIV picking their ART within the clinic. The high MMT retention rate was in contrast to earlier studies in developed and still developing countries [22, 23]. Our findings could be explained by the existing collaboration between the Kisauni MMT staff and CSO’s monthly defaulter/LTFU follow-up mechanism as well as the open weekday re-induction strategy. Outreach Workers at the two CSOs use the Kisauni MAT clinic health records information officer (HRIO) to identify defaulters and LTFUs, who are subsequently tracked and re-inducted back to the MMT program. Despite the higher retention rate, significant factors associated with LTFU outcome were the lack of formal education, unemployment, and being a non-injecting drug user at enrolment.

The higher HIV and viral hepatitis prevalence found in this study were possibly a consequence of the concurrent risky sexual behaviour of opioid-dependent persons and the sharing of needles and syringes during the drug use period before joining MMT [24]. The higher rate of HIV and other infectious diseases among females in this study is not unexpected; it has been observed in both industrialised and developing countries. It has been proven that owing to drug use, females might engage in sex work and have multiple sexual partners to finance their addiction [25, 26]. Moreover, females and young opioid users increase their vulnerability to HIV by starting to inject and share needles and syringes with their peers [27, 28]. However, our findings on HCV prevalence among MMT clients contrasted with a Kenyan study on active drug users, which reported a higher prevalence of above 50% [13].

Incidences of new HIV and HCV infections were documented among the enrolled MMT clients, which is consistent with earlier studies among MMT clients globally [6, 29]. It was worth noting that all HIV-positive clients including the new cases were linked to a Comprehensive Care Clinic (CCC) at the clinic or nearby health facilities and were receiving Antiretroviral (ART) medication. The majority of them had undetectable viral loads, implying that supervised ART with methadone intake enhances HIV treatment adherence and outcomes. These findings are consistent with prior research in Nairobi and Tanzania, which reported improved ART adherence among opioid-dependent clients on methadone [7, 30]. However, when compared with the total number of opioid users living with HIV enrolled in the Kisauni MAT clinic, the proportion of these clients taking ART at the Kisauni Clinic CCC was low.

We found a connection between LTFU and the lack of formal education among the MMT clients. This result contrasted with those from Yunnan, China, which found no link between education level and LTFU when taking MMT [6]. Clients’ lack of primary school education implies a potential lack of employability skills which might hamper their reemployment during recovery and thus could not finance their daily travel costs. We observed a small percentage of working MMT clients which concurs with another study in Dar es Salaam, Tanzania under similar settings which reported a 17% employment rate, [31]. Interestingly, the unemployed clients in this study had higher odds of being LTFU to the program than those employed. The restricted morning service delivery model for Daily Observed Therapy (DOT) adopted in the clinic may have deterred clients from job-seeking since employed clients are unable to work regular hours before picking up their dose. The strategy might have created the notion that MMT clients are still active drug users, thereby rendering it difficult for them to be employed and fund their daily commuter costs to the clinic. Consequently, it’s plausible that these clients spend a significant amount of time in drug consumption spaces maintaining connections with persons who are actively using drugs, thereby increasing their dropout risks.

Numerous research, including those conducted by Ledgerwood and Nordt et. al, have explored the impact of the mode of heroin administration on treatment outcomes, however, they did not find any significant effect [23, 32, 33]. After 2 years of follow-up, our study discovered a level of significance in non-injectors dropping out of methadone treatment. Unlike non-injectors who drop out of the MMT program, IDUs are likely to be HIV positive, which may increase their timely health-seeking behaviour and adherence to scheduled clinical reviews and psychological support. These findings add to the evidence presented by Morgan et al in South Africa, who found that non-injectors had poorer treatment outcomes than injectors in inpatient rehabilitation [34]. Despite the similarity in this finding, the study by Morgan et al was among persons with OUD in an inpatient facility.

This study was not without limitation since we based our findings on the data captured in the registers in the clinic. Such records are prone to errors due to incomplete or incorrect data entry by overburdened health workers, inaccurate self-reports of MMT clients, and weak supervision of MMT services. Missing or tattered health records due to sub-optimal filing and archiving could have compromised some study findings. According to the facility, a small percentage of the clients did not retest for urine toxicology, HCV and HBV after joining the program due to commodity stock-outs; therefore, the proportion of polydrug use and clients seroconverting while on MMT may have been underestimated.

Despite the above-mentioned limitations, reported results are credible as the data was drawn from complementary data sources, i.e., client files and cards compared with facility registers and Excel Tools from multiple service delivery points. Our findings concur with previous studies in Kenya and elsewhere while revealing new knowledge on the vulnerability of female opioid-dependent persons to MMT attrition despite being younger and more educated.

### Conclusion

Our study has confirmed the effectiveness of a low threshold One Stop Shop Methadone maintenance treatment model that integrates HIV, viral hepatitis and STI prevention and treatment interventions for opioid-dependent persons in Mombasa, Kenya. However, to improve treatment adherence and outcomes and avert the perception of MMT as simply “Replacing a Drug with a Drug,” a more patient-centred approach is needed that can increase demand and retention for MMT by other persons with opioid use disorders. This may include refresher training, alternative dispensing models for MMT, and providing short treatment breaks and take-home doses as incentives for stable and drug-free clients. Finally, there is a need to integrate CCC into MMT services, and further research on evidence-based approaches for addressing HIV acquisition, and vulnerability of females and young people with opioid use disorders.

## Data Availability

The raw data supporting the conclusion of this article will be made available by the authors, without undue reservation.

## References

[1] United Nations Office on Drugs and Crime. World drug report 2018 (2018).

[2] Degenhardt L Grebely J Stone J Hickman M Vickerman P Marshall BDL Global patterns of opioid use and dependence: harms to populations, interventions, and future action. Lancet (London, England) (2019) 394(10208):1560–79. 10.1016/S0140-6736(19)32229-9 31657732 PMC7068135

[3] Kurth A Cherutich P Conover R Chhun N Bruce RD Lambdin BH. The opioid epidemic in Africa and its impact. Springer Link (2018) 176(1):428–53. 10.1007/s40429-018-0232-9 PMC7269163 32494564

[4] Bertschy G. Methadone maintenance treatment: an update. Eur Arch Psychiatry Clin Neurosci (1995) 245(2):114–24. 10.1007/BF02190738 7654787

[5] WHO. Guidelines for the psychosocially assisted pharmacological treatment of opioid dependence [Internet]. World Health Organization (2009). Available from: https://www.who.int/publications-detail-redirect/9789241547543. 23762965

[6] Chang Y-P Duo L Kumar AMV Achanta S Xue H-M Satyanarayana S Retention and HIV seroconversion among drug users on methadone maintenance treatment in Yunnan, China. Public Health Action (2014) 4(1):28–34. 10.5588/pha.13.0101 26423758 PMC4479105

[7] Mbogo LW Sambai B Monroe-Wise A Ludwig-Barron NT Guthrie BL Bukusi D Participation in methadone programs improves antiretroviral uptake and HIV viral suppression among people who inject drugs in Kenya. J Substance Abuse Treat (2021) 134:108587. 10.1016/j.jsat.2021.108587 PMC11225265 34391587

[8] WHO. WHO/UNODC/UNAIDS position paper : substitution maintenance therapy in the management of opioid dependence and HIV/AIDS prevention/ World Health Organization, United Nations Office on Drugs and Crimes, UNAIDS [Internet] (2023). Available from: https://www.who.int/publications-detail-redirect/who-unodc-unaids-position-paper.-substitution-maintenance-therapy-in-the-management-of-opioid-dependence-and-hiv-aids-prevention .

[9] Ministry of Health. Ministry of health republic of Kenya the national protocol for treatment of substance use disorders in Kenya (2017). p. 3–7.

[10] NACC. Final report republic of Kenya (2018). Available from: www.nacc.or.ke.

[11] Docslib. Background medically assisted therapy (MAT) in Kenya MAT [Internet] (2024). Available from: https://docslib.org/doc/13558854/background-medically-assisted-therapy-mat-in-kenya-mat .

[12] NASCOP. Reaching the unreached the evolution of Kenya’s HIV/AIDS prevention programme for key populations (2016). p. 1–82.

[13] Akiyama MJ Cleland CM Lizcano JA Cherutich P Kurth AE. Prevalence, estimated incidence, risk behaviours, and genotypic distribution of hepatitis C virus among people who inject drugs accessing harm-reduction services in Kenya: a retrospective cohort study. Lancet Infect Dis (2019) 19(11):1255–63. 10.1016/S1473-3099(19)30264-6 31540840 PMC7099605

[14] Oguya FO Kenya PR Ongecha F Mureithi P Musyoka H Muraguri N Rapid situational assessment of people who inject drugs (PWID) in Nairobi and coastal regions of Kenya: a respondent driven sampling survey. BMC Public Health (2021) 21(1):1549. 10.1186/s12889-021-11373-9 34391389 PMC8364050

[15] Rhodes T Guise A Ndimbii J Strathdee S Ngugi E Platt L Is the promise of methadone Kenya’s solution to managing HIV and addiction? A mixed-method mathematical modelling and qualitative study. BMJ Open (2015) 5(3):e007198. 10.1136/bmjopen-2014-007198 PMC4360822 25748417

[16] Ngarachu EW Kiburi SK Owiti FR Kangethe RN. Cannabis use among patients attending a methadone maintenance treatment clinic in Nairobi, Kenya elizabeth. J Chem Inf Model (2019) 53(9):1689–99. 10.1186/s13011-022-00437-7

[17] Mogaka B Kiburi SK Mutinda M Kendagor M. Estimate cost of providing methadone maintenance treatment at a methadone clinic in Nairobi Kenya: direct costs. Pan Afr Med J (2021) 38(84):84–6. 10.11604/pamj.2021.38.84.21991 33889250 PMC8033195

[18] Wanjihia V Muniu E Mwangi M Mwangi C Mutisya R Ndemwa P Comparison of nutritional status and food insecurity among people who inject illicit drugs, non drug users and those on methadone treatment in selected areas of Nairobi, Kenya. J Alcohol Drug Dependence (2018) 06(05). 10.4172/2329-6488.1000319

[19] Chen Z Tang X Xu C Wang C Ling L. Exploring factors jointly associated with recurrent relapse and dropout of methadone maintenance treatment clients in Guangdong, China: a retrospective cohort study. Drug and Alcohol Dependence (2023) 243:109739. 10.1016/j.drugalcdep.2022.109739 36535097

[20] Zhang L Zou X Zhang D Li X Zhao P Ling L. Investigation of repeat client drop-out and Re-enrolment cycles in fourteen methadone maintenance treatment clinics in guangdong, China. PLOS ONE (2015) 10(10):e0139942. 10.1371/journal.pone.0139942 26484772 PMC4618733

[21] WHO. Regional Office for Africa. The national protocol for treatment of substance use disorders in Kenya [Internet]. WHO Regional Office for Africa (2024). Available from: https://www.afro.who.int/publications/national-protocol-treatment-substance-use-disorders-kenya.

[22] Cox J Allard R Maurais E Haley N Small C. Predictors of methadone program non-retention for opioid analgesic dependent patients. J Substance Abuse Treat (2013) 44(1):52–60. 10.1016/j.jsat.2012.03.002 22538172

[23] Darke S Ross J Teesson M. Twelve-month outcomes for heroin dependence treatments: does route of administration matter? Drug Alcohol Rev (2005) 24(2):165–71. 10.1080/09595230500102657 16076586

[24] Degenhardt L Charlson F Stanaway J Larney S Alexander LT Hickman M Estimating the burden of disease attributable to injecting drug use as a risk factor for HIV, hepatitis C, and hepatitis B: findings from the Global Burden of Disease Study 2013. The Lancet (2016) 16(12):1385–98. 10.1016/S1473-3099(16)30325-5 27665254

[25] Lambdin BH Bruce RD Chang O Nyandindi C Sabuni N Zamudio-Haas S Identifying programmatic gaps: inequities in harm reduction service utilization among male and female drug users in dar es Salaam, Tanzania. PLOS ONE (2013) 8(6):e67062. 10.1371/journal.pone.0067062 23825620 PMC3692420

[26] Nsanzimana S Mills EJ Harari O Mugwaneza P Karita E Uwizihiwe JP Prevalence and incidence of HIV among female sex workers and their clients: modelling the potential effects of intervention in Rwanda. BMJ Glob Health (2020) 5(8):e002300. 10.1136/bmjgh-2020-002300 PMC7412619 32764126

[27] Baluku M Wamala T. When and how do individuals transition from regular drug use to injection drug use in Uganda? Findings from a rapid assessment. Harm Reduction J (2019) 16:73. 10.1186/s12954-019-0350-2 PMC6929349 31870396

[28] Clark M Buchanan R Kovensky R Leve LD. Partner influences on young women’s risky drug and sexual behavior. Reprod Health (2018) 15(1):156. 10.1186/s12978-018-0598-0 30219076 PMC6139176

[29] Gibson DR Flynn NM McCarthy JJ. Effectiveness of methadone treatment in reducing HIV risk behavior and HIV seroconversion among injecting drug users. AIDS (1999) 13(14):1807–18. 10.1097/00002030-199910010-00002 10513638

[30] Saleem HT Knight D Yang C Kidorf M Latkin C Nkya IH. HIV Stigma, HIV status disclosure, and ART adherence in the context of an integrated opioid use disorder and HIV treatment setting in Dar es Salaam, Tanzania. AIDS Care (2023) 35(1):91–4. 10.1080/09540121.2022.2032575 35109727 PMC9343474

[31] Ubuguyu O Tran OC Bruce RD Masao F Nyandindi C Sabuni N Improvements in health-related quality of life among methadone maintenance clients in Dar es Salaam, Tanzania. Int J Drug Pol (2016) 30:74–81. 10.1016/j.drugpo.2016.03.005 PMC4829437 27017376

[32] Ledgerwood DM Lister JJ LaLiberte B Lundahl LH Greenwald MK. Injection opioid use as a predictor of treatment outcomes among methadone-maintained opioid-dependent patients. Addict Behaviors (2019) 90:191–5. 10.1016/j.addbeh.2018.10.046 30412910

[33] Nordt C Vogel M Dürsteler KM Stohler R Herdener M. A comprehensive model of treatment participation in chronic disease allowed prediction of opioid substitution treatment participation in Zurich, 1992–2012. J Clin Epidemiol (2015) 68(11):1346–54. 10.1016/j.jclinepi.2015.05.002 26073899

[34] Morgan N Daniels W Subramaney U. Smoking heroin with cannabis versus injecting heroin: unexpected impact on treatment outcomes. Harm Reduction J (2019) 16(1):65. 10.1186/s12954-019-0337-z PMC6896288 31805971

